# Targeted delivery by pH-responsive mPEG-S-PBLG micelles significantly enhances the anti-tumor efficacy of doxorubicin with reduced cardiotoxicity

**DOI:** 10.1080/10717544.2021.2008052

**Published:** 2021-11-29

**Authors:** Qiyi Feng, Junhuai Xu, Xinyi Liu, Haibo Wang, Junjie Xiong, Kai Xiao

**Affiliations:** a Precision Medicine Research Center & Sichuan Provincial Key Laboratory of Precision Medicine and National Clinical Research Center for Geriatrics, West China Hospital, Sichuan University, Chengdu, China; b National Chengdu Center for Safety Evaluation of Drugs, West China Hospital, Sichuan University, Chengdu, China; c College of Biomass Science and Engineering, Sichuan University, Chengdu, China; d Department of Pancreatic Surgery, West China Hospital, Sichuan University, Chengdu, China

**Keywords:** Tumor microenvironment, pH-responsive, micelles, β-thiopropionate linkage, drug delivery

## Abstract

Stimuli-responsive nanotherapeutics hold great promise in precision oncology. In this study, a facile strategy was used to develop a new class of pH-responsive micelles, which contain methoxy polyethylene glycol (mPEG) and poly(carbobenzoxy-l-glutamic acid, BLG) as amphiphilic copolymer, and β-thiopropionate as acid-labile linkage. The mPEG-S-PBLG copolymer was synthesized through one-step ring-opening polymerization (ROP) and thiol-ene click reaction, and was able to efficiently encapsulate doxorubicin (DOX) to form micelles. The physicochemical characteristics, cellular uptake, tumor targeting, and anti-tumor efficacy of DOX-loaded micelles were investigated. DOX-loaded micelles were stable under physiological conditions and disintegrated under acidic conditions. DOX-loaded micelles can be internalized into cancer cells and release drugs in response to low pH in endosomes/lysosomes, resulting in cell death. Furthermore, the micellar formulation significantly prolonged the blood circulation, reduced the cardiac distribution, and selectively delivered more drugs to tumor tissue. Finally, compared with free DOX, DOX-loaded micelles significantly improved the anti-tumor efficacy and reduced systemic and cardiac toxicity in two different tumor xenograft models. These results suggest that mPEG-S-PBLG micelles have translational potential in the precise delivery of anti-cancer drugs.

## Introduction

Tumor microenvironment (TME) is a complex physical and biochemical system, which plays a critical role in the initiation, progression, metastasis, and drug resistance of tumors (Quail & Joyce, 2013; Wu & Dai, 2017). It is well known that TME has unique biological characteristics different from normal tissues, such as acidic pH, hypoxia, elevated glutathione levels, and up-regulation or down-regulation of certain enzymes (Maacha et al., 2019). These intrinsic features of TME have been utilized to develop stimuli-responsive nanoparticles (NPs) for the precise drug delivery, which is of great significance to improve anti-tumor efficacy and reduce systemic toxicity. Among these biological stimuli, pH-responsiveness has gained significant attention and has been extensively studied. Acidic extracellular conditions (pH 6.5–6.8) caused by anaerobic glycolysis in hypoxic TME are considered as a major hallmark of tumor tissues (Karimi et al., 2016; Huber et al., 2017), which makes pH value a suitable stimulus for tumor-specific and controllable drug release. In addition, some pH-responsive NPs have also shown the ability to disrupt the membrane of endosomes/lysosomes (pH 5–6), ensuring more efficient drug delivery to the site of action, such as cytosol (Wu et al., 2018). Studies have shown that pH-responsive NPs have favorable safety profile, controlled drug release, and improved anti-tumor efficacy than those without pH response (Chen et al., 2019; Jia et al., 2020). Based on the reported pH-responsive NPs, there are usually two different preparation methods. One method is to introduce the pH-responsive linkages into the polymer chain, and the other is to design a polymer containing an ionizable groups (Nguyen et al., 2018). However, most of the pH-sensitive bonds, such as acetal/ketal, hydrazone, orthoester, Schiff base, and cis-aconityl, are not stable enough in blood circulation and exhibit relatively rapid drug release behavior *in vivo* (Li et al., 2015; Sonawane et al., 2017; Huang et al., 2018; Zhai et al., 2018; Wang et al., 2019). Besides, some of these pH-responsive NPs have complex preparation processes that limit their large-scale production, reproducibility, and clinical translation. Therefore, it remains challenging to construct NPs with relatively simple synthesisprocess, good biocompatibility, high blood stability, and pH-responsiveness.

Recently, click reaction has become a widely used method for controlled polymerization (Hoyle & Bowman, 2010). Notably, the thiol-ene click reaction is an efficient green reaction because it does not require metallic catalyst (Zou et al., 2018). Compared with other pH-sensitive linkages, β-thiopropionate linkage is relatively stable at neutral pH but slowly hydrolyzed in acidic pH conditions driven by the generation of a partial positive charge on the ester carbonyl carbon owing to the inductive effect of sulfur atom (Qiu et al., 2016). This controlled drug release manner makes the β-thiopropionate linkage a suitable pH-responsive ‘ON/OFF’ switch to realize the sustained drug release (Hoyle & Bowman, 2010). More importantly, the hydrolysis rate of β-thiopropionate could be controlled by simply adjusting the number of methylene units, such as the length of a sulfide and it is facile to introduce β-thiopropionate linkage in the polymer by a thiol-ene click reaction under mild reaction conditions (Hoyle & Bowman, 2010). For example, Dan & Ghosh (2013) prepared an amphiphilic triblock copolymer by sequential thiol-acrylate Michael addition reaction in one pot, and the resulting polymersomes assembled by the copolymer exhibited pH-specific sustained drug release properties.

**Figure 1.**
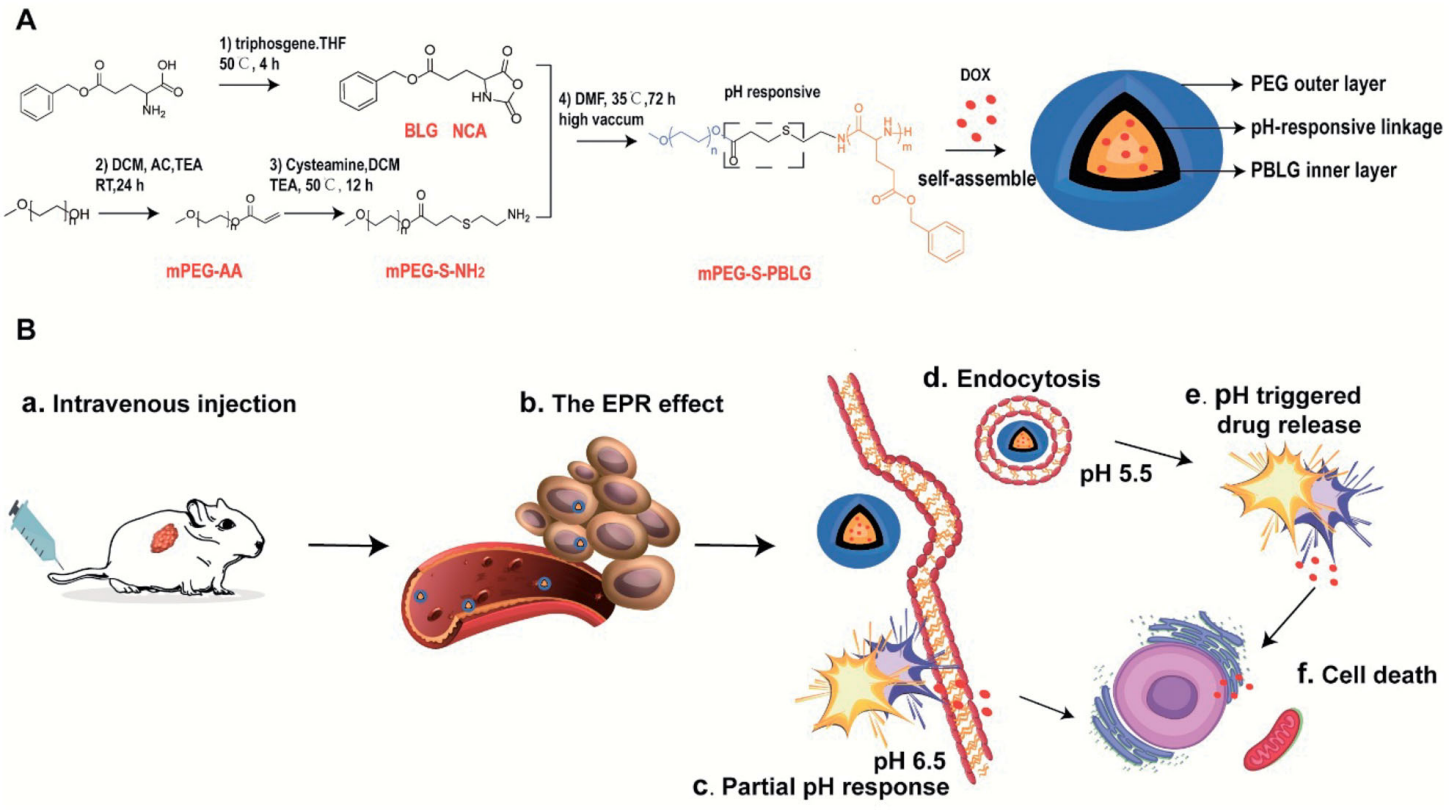
Chemical structure of mPEG-S-PBLG copolymer and schematic diagram of the resulting self-assembled pH-responsive micelles for efficient delivery of DOX in targeted cancer therapy. (A) The amphiphilic polymer is able to efficiently encapsulate chemotherapeutic drugs (such as DOX) to form micellar formulation. (B) After intravenous injection (a), DOX-loaded micelles could accumulate at tumor sites through the EPR effect (b) and responsively release drugs in the mild acidic extracellular environment (pH 6.5, c) or the acidic intracellular compartment (endosome/lysosome, pH 5.5, d) upon the internalization into cancer cells (e), resulting in cancer cell death (f).

Polymeric micelles have unique core–shell structure and are usually formed by amphiphilic block copolymers (BCPs), which can improve the solubility of insoluble drugs and protect them from degradation and metabolism (Ke et al., 2014; Zhang et al., 2017). As the biocompatibility of polymers directly affects their application as biomedical materials, the use of biodegradable materials for polymerization has become an effective method to construct biocompatible polymers. Polypeptides, which have the same composition as protein and good natural biocompatibility, are stable against hydrolysis and can be rapidly degraded into α-amino acids *in vivo* under the catalysis of specific enzymes (Lu et al., 2020). Block copolymers with controllable degree of polymerization and molecular structure can be easily obtained by the ring-opening polymerization (ROP) of α-amino acid N-carboxyanhydride (NCA) initiated by macromolecules with an amino terminus (Mai & Eisenberg, 2012; Gonzalez-Henriquez et al., 2017). Herein, we report a new type of pH-responsive micellar NPs based on amphiphilic BCP comprising hydrophilic methoxy polyethylene glycol (mPEG) and hydrophobic poly(N-carbobenzoxy-l-glutamic acid) (PBLG) for sustained and targeted delivery of anti-cancer chemotherapeutic drugs. mPEG, as a hydrophilic group, is widely attached to the periphery of polymers and forms a hydrated shell on the surface of micelles, thus protecting the interaction between the micelles and plasma proteins (Zaman et al., 2019). Carbobenzoxy-l-glutamic acid is a unique building block for the polymer construction owing to its advantages of convenient synthesis, simple purification process, and nontoxicity. The polymer PBLG is known to adopt an α-helical conformation under various conditions, thus allowing acceleration of the polymerization through interhelix cooperative macrodipole interactions (Lv et al., 2020). The acid labile β-thiopropionate linkage was introduced in the backbone of mPEG-PBLG copolymer through thiol-ene click reaction, endowing the copolymer with pH-responsive capacity. The resulting amphiphilic copolymer (mPEG-S-PBLG) can self-assemble into micelles with core/shell structure in aqueous solution, and efficiently encapsulate therapeutic drugs such as doxorubicin (DOX) in their core. Once drug-loaded micelles accumulate at tumor sites through the enhanced permeability and retention (EPR) effect and are exposed to the acidic extracellular conditions in TME or acidic compartments within cancer cells (endosomes, lysosomes), the pH-responsive cleavage of β-thiopropionate bonds will induce the gradual disintegration of mPEG-S-PBLG micelles, leading to specific and sustained drug delivery to tumor tissue and cells (Figure 1 and Figure S1). The physicochemical properties of mPEG-S-PBLG micelles including the critical micelle concentration (CMC), morphology, drug loading capacity, and pH-responsive drug release were fully characterized. Then, the uptake, cytotoxicity and biocompatibility of DOX-loaded micelles in cancer cells (U-87 MG human glioblastoma cells and SK-HEP-1 human hepatocarcinoma cells), as well as their pharmacokinetics, biodistribution, and tumor targeting capability in normal and U-87 MG tumor bearing mice were investigated. Finally, the anti-tumor efficacy and toxicity profiles of DOX-loaded micelles were evaluated in both U-87 MG and SK-HEP-1 tumor xenograft models when compared with free DOX. The results demonstrated that mPEG-S-PBLG micelles could efficiently encapsulate DOX, specifically deliver DOX to the tumor sites, and programmatically release the drug in response to acidic TME. As expected, this novel micellar formulation improved the anti-tumor efficacy of DOX, and reduced its systemic and cardiac toxicity, which provides a strategy to solve the dilemma of chemotherapeutics in solid tumors.

## Materials and methods

### Materials

The mPEG_4000_ was purchased from Best-reagent (Beijing, China). N-Carbobenzoxy-l-glutamic acid (PBLG) was purchased from Wuxi Asiapeptide Biotechnology Co., Ltd. (Wuxi, China). Acryloyl chloride, cysteamine hydrochloride, and DOX·HCl were purchased from Macklin (Shanghai, China). 4,6-Diamidino-2-phenylindole (DAPI), 1,10-dioctadecyl-3,3,30,30 tetramethylindodicarbocyanine perchlorate (DiD), and LysoTracker Green were purchased from Invitrogen (Carlsbad, CA). Cell Counting Kit-8 (CCK-8) was purchased from Dojindo (Kumamoto, Japan). Hoechst 33342 was purchased from BD Biosciences (San Jose, CA).

### Synthesis of acrylate-mPEG

mPEG_4000_ (20 g, 0.005 mol) was dissolved in a round bottom flask with dichloromethane (DCM) (50 mL), followed by the addition of triethylamine (TEA) (2 g, 0.02 mol) as acid binding agent. With vigorous stirring and ice bath, acryloyl chloride (1.62 mL, 0.02 mol) in DCM (20 mL) was added into the flask dropwise, the reaction mixture was stirred for 24 h at room temperature. After the reaction was completed, the mixture was washed three times with NaHCO_3_ aqueous solution and saturated brine, dried with anhydrous Na_2_SO_4_, most of the solvent was removed by evaporation, and then precipitation in the anhydrous diethyl ether to obtain the white powder product of acrylate-mPEG. The yield was 76% (Luo et al., 2020).

### Synthesis of mPEG-S-NH_2_

Acrylate-mPEG (4.06 g, 0.001 mol), cysteamine hydrochloride (0.15 g, 0.0013 mol), DCM (30 mL), and TEA (0.1 g, 0.001 mol) were added in a 100 mL flask. The reaction was kept at 50 °C for 12 h. The final solution was washed three times with NaHCO_3_ aqueous solution and saturated brine, dried with anhydrous Na_2_SO_4_. The concentrated solution was added dropwise to excessive diethyl ether to obtain the product mPEG-S-NH2, a white powder with an isolated yield of 83.2% (Krasznai et al., 2012).

### Synthesis of mPEG-S-PBLG

mPEG-S-PBLG was synthesized by sequential ROP of BLG-NCA monomers in DMF using mPEG-S-NH_2_ as an initiator. Under a nitrogen atmosphere, BLG NCA (0.3 g, 1.14 mmol), mPEG-S-NH_2_ (0.3 g, 0.073 mmol, monomer to initiator ratio of 16), and DMF (8 mL) were added to a 50 mL flask. The reaction kept at 35 °C for another 72 h. The copolymer was precipitated in excess diethyl ether, purified by dissolving in DCM, then reprecipitated twice with diethyl ether, dried in vacuum at room temperature for 24 h. The yield was 72.3% (Wu et al., 2011).

### Characterization of polymers

^1^H NMR spectra were recorded on a Bruker AV 400 spectrometers (Billerica, MA) with CDCl_3_ as solvent. Fourier transform-infrared (FTIR) spectrometer (Nicolet 560 infrared spectrometer) was used to record FTIR spectra at 25 °C, and the transmittance mode was used. The CMC was determined by a fluorescence spectrophotometer (Perkin Elmer, LS-55, Waltham, MA) using pyrene as fluorescence probe according to previous reports (Yan et al., 2020).

### Preparation of blank micelles

mPEG-S-PBLG micelles were prepared by a dialysis method. mPEG-S-PBLG copolymer (10 mg) was dissolved in DMF (1.0 mL) and stirred vigorously at room temperature. Deionized water (10 mL) was dropwise added in the solution, stirred for 5 h, and then dialyzed against deionized water in a dialysis bag with a molecular weight cutoff (MWCO) of 3500 Da for 48 h to obtain blank micelles with a concentration of 1.0 mg mL^−1^. Subsequently, the solution was filtered through 0.22 μm pore-sized microporous membrane. The micelles solution was lyophilized and stored at −20 °C.

### Preparation and physicochemical characterization of DOX-loaded micelles

DOX-loaded micelles were prepared by dialysis method as described above. Briefly, mPEG-S-PBLG (20 mg) and DOX·HCl (4 mg) were dissolved in DMF (2 mL), and a drop of TEA was added into the solution and stirred overnight at room temperature. Then, deionized water (20 mL) was dropwise added, and the solution was dialyzed against deionized water (MWCO 3500 Da) for 48 h. The resulting DOX-loaded micelles was lyophilized and stored at −20 °C. In order to track their fate *in vivo*, the hydrophobic NIRF dye DiD was encapsulated into the micelles using the same method. According to the standard curve of DOX, the drug-loading efficiency (DLE) and drug-loading content (DLC) of micelles were determined by UV spectrum (Analytic-jena Specord, S600, Upland, CA) and calculated by the following equations:

$$\begin{array}{l} \text{DLC}\%=\text{weight of drug in micelles}\\ \text{ /weight of drug-loaded micelles}\times \text{1}00\%\\ \text{DLE }\%=\text{weight of drug in micelles}\\ \text{ /total weight of loaded drug}\times \text{1}00\% \end{array}$$

The morphology and particle size distribution of DOX-loaded micelles were characterized by transmission electron microscopy (TEM, JEOL JEM-1200EX, Tokyo, Japan) and dynamic light scattering (DLS, Malvern, Zetasizer Nano ZPS, Malvern, UK), respectively. In addition, the change in particle size and morphology of mPEG-S-PBLG micelles was determined by DLS and TEM under different pH conditions (7.4, 6.5, and 5.0). To investigate pH-triggered cleavage of β-thiopropionate bonds, the micelles were dialyzed in an acidic (pH 5.0) aqueous solution for two days and the precipitate in the dialysis bag was detected by ^1^H NMR.

The drug release profiles from DOX-loaded micelles were measured by the dialysis method as described previously (Yan et al., 2020). In brief, aliquots of DOX-loaded micelles solution were added into dialysis cartridges with the MWCO of 3500 Da, and dialyzed against PBS with different pH values (7.4, 6.5, and 5.0) under the condition of continuous shaking at 100 rpm. At predetermined time interval, the remaining DOX concentration in the dialysis cartridge was measured by the UV absorption at 500 nm.

### Cell culture and animals

Human glioblastoma cell lines (U-87 MG), hepatocellular carcinoma cell line (SK-HEP-1), and rat cardiac myocytes (H9C2) were purchased from Chinese Academy of Science Cell Bank for Type Culture Collection (Shanghai, China). These cells were cultured in RPMI-1640 or DMEM medium supplemented with 10% fetal bovine serum (FBS), 100 U/mL penicillin G, and 100 μg mL^−1^ streptomycin at 37 °C in a humidified 5% CO_2_ incubator.

Female BALB/c mice and BALB/c nude mice (6–8 weeks old) were obtained from Beijing Vital River Laboratory Animal Technology (Beijing, China). All animals were kept under specific pathogen-free (SPF) conditions with a regular 12 h light–dark cycle and had free access to water and feed. All animal experiments were performed in accordance with the Association for Assessment and Accreditation of Laboratory Animal Care (AAALAC) guidelines and the protocols approved by the Institutional Animal Care and Use Committee (IACUC) of West China Hospital, Sichuan University.

### Cellular uptake

The cells were seeded onto confocal dishes at a density of 2–3 × 10^4^ cells. After 24-h incubation, free DOX or DOX-loaded micelles were added into the medium at the final DOX concentration of 5 μM, and incubated for 6 h or 18 h at 37 °C. The cells were washed three times with cold saline and fixed with 75% ethanol for 15 min at room temperature. The nuclei were stained with DAPI and the cellular uptake of DOX was visualized under confocal laser scanning microscopy (CLSM; IX83, Olympus, Tokyo, Japan). In order to detect the subcellular localization, free DOX and DOX-loaded micelles were added in the medium and incubated for 6 h at 37 °C, and then the cells were stained with LysoTracker Green (200 nM) for 2.5 h and Hoechst 33342 (10 μg mL^−1^) for another 1 h. After removing the medium, the living cells were washed three times with cold saline and directly observed with CLSM.

To visualize the pH-responsive drug release upon cellular uptake, U-87 MG cells were incubated with free DOX or DOX-loaded micelles in culture medium with different pH values (7.4 or 6.5) at 37 °C for 6 h. Then, cells were washed with cold saline and fixed with 75% ethanol, stained with DAPI, and observed under CLSM. The cellular uptake of U-87 MG cells at different pH value was also quantitatively analyzed by flow cytometry (ACEA NovoCyte, Agilent, Santa Clara, CA).

The cell uptake of free DOX and DOX-loaded micelles was quantitatively analyzed by flow cytometry. Cells were incubated with free DOX or DOX-loaded micelles at different DOX concentrations (1, 2.5, and 5 μM) for 6 h and 18 h at 37 °C, respectively. Then, cells were collected and resuspended in PBS for flow cytometry analysis (ACEA NovoCyte, Agilent, Santa Clara, CA).

### *In vitro* cytotoxicity assay

Cells were seeded in 96-well plates at a density of 3–4 × 10^3^ cells/well and incubated for 24 h. Cells were incubated with different concentrations of free DOX and DOX-loaded micelles, as well as the equivalent concentration of blank micelles. After 72 h, CCK-8 solution was added to each well according to the manufacturer's instructions and incubated for another 1 h. The absorbance at 450 nm and 600 nm was detected using a microplate ELISA reader (Epoch, BioTek, Winooski, VT). Results were shown as the average cell viability ((OD_treat_ – OD_blank_)/(OD_control_ – OD_blank_)×100%) of triplicate wells.

### Pharmacokinetics and biodistribution studies

DOX·HCl and DOX-loaded micelles were intravenously injected into normal female BALB/c mice at a dose of 10 mg kg^−1^ body weight (*n* = 5). Blood was collected at different time points (5 min, 10 min, 30 min, 1 h, 2 h, 4 h, 8 h, and 24 h) post injection. The blood was centrifuged to obtain plasma for the analysis. DOX was extracted from plasma by mixing 20 μL plasma with 100 μL methanol, sonication for 4 min, and centrifugation at 15,000 rpm for 10 min. The supernatant (100 μL) was collected and added into 96-well plates with black bottom. The fluorescence (ex/em: 470/590 nm) of the supernatant was obtained using a microplate reader (Epoch, BioTek, Winooski, VT) and the concentration of DOX was calculated by the standard curve. The standard curve of DOX in plasma was generated by adding different concentrations of free DOX into plasma followed by the same extraction and determination method. Non-compartmental pharmacokinetic analysis was performed and pharmacokinetic parameters were calculated using Phoenix WinNonlin 6.4 software. The mice were sacrificed 24 h post injection and major organs (heart, liver, spleen, lung, and kidney) were excised to evaluate the tissue distribution of DOX. One hundred milligrams tissues were mixed with 900 μL extraction buffer (10% Triton X-100, deionized water, and isopropanol with 1:2:15 volumetric ratio), homogenized and DOX was extracted overnight at −20 °C. The samples were centrifuged at 3000 rpm for 5 min and the supernatant was collected for the fluorescence measurement. The standard curve of DOX in each tissue was generated by mixing different concentrations of free DOX with each tissue homogenate. The biodistribution data were expressed as the % injected dose per gram tissue (ID/g).

### NIRF optical imaging

The tumor model was established by subcutaneous injection of 1 × 10^6^ U-87 MG cells into the right flank of BALB/c nude mice. One hundred and fifty microliters NIRF dyes DiD and DiD labeled-micelles (at the DiD concentration of 0.25 mg mL^−1^) were intravenously injected into tumor bearing mice, respectively (*n* = 3). Mice were anesthetized and scanned using the IVIS imaging system (Lumina III, PerkinElmer, Rodgau, Germany) at different time points (1 h, 2 h, 8 h, 24 h, and 48 h). Animals were euthanized 48 h post-injection and major organs were excised for *ex vivo* optical images and quantitative fluorescence intensity analysis.

### *In vivo* therapeutic study

The mouse models of glioblastoma (U-87 MG) and hepatocarcinoma (SK-HEP-1) were established respectively to evaluate the anti-tumor efficacy and safety of DOX-loaded micelles. When tumor volume reached 100–300 mm^3^, mice were randomly divided into three groups (*n* = 5) and intravenously injected with saline, free DOX, DOX-loaded micelles every other day for a total of seven doses. The doses of free DOX and DOX-loaded micelles were the same, at 5 mg kg^−1^ and 2.5 mg kg^−1^ respectively in glioblastoma and hepatocarcinoma models. Tumor volume and body weight were measured every three days. The tumor size larger than 1000 mm^3^ was considered as the end point of survival data. At the end of the experiment, blood samples were collected for the measurement of serum chemistry including alanine aminotransferase (ALT), aspartate aminotransferase (AST), blood urea nitrogen (BUN), creatine kinase (CK), and lactate dehydrogenase (LDH). The tumor and major organs were also excised for weighing and histopathological examination. The tumor section was also stained with TUNEL (apoptosis marker) and Ki67 (proliferation marker) for immunohistochemical analysis.

### Statistical analysis

All data were expressed as the mean ± standard deviation (SD) unless otherwise indicated, and analyzed using SPSS software (version 20.0, Chicago, IL). A value of *p*< .05 was considered statistically significant.

## Results

### Synthesis and characterization of mPEG-S-PBLG

**Figure 2.**
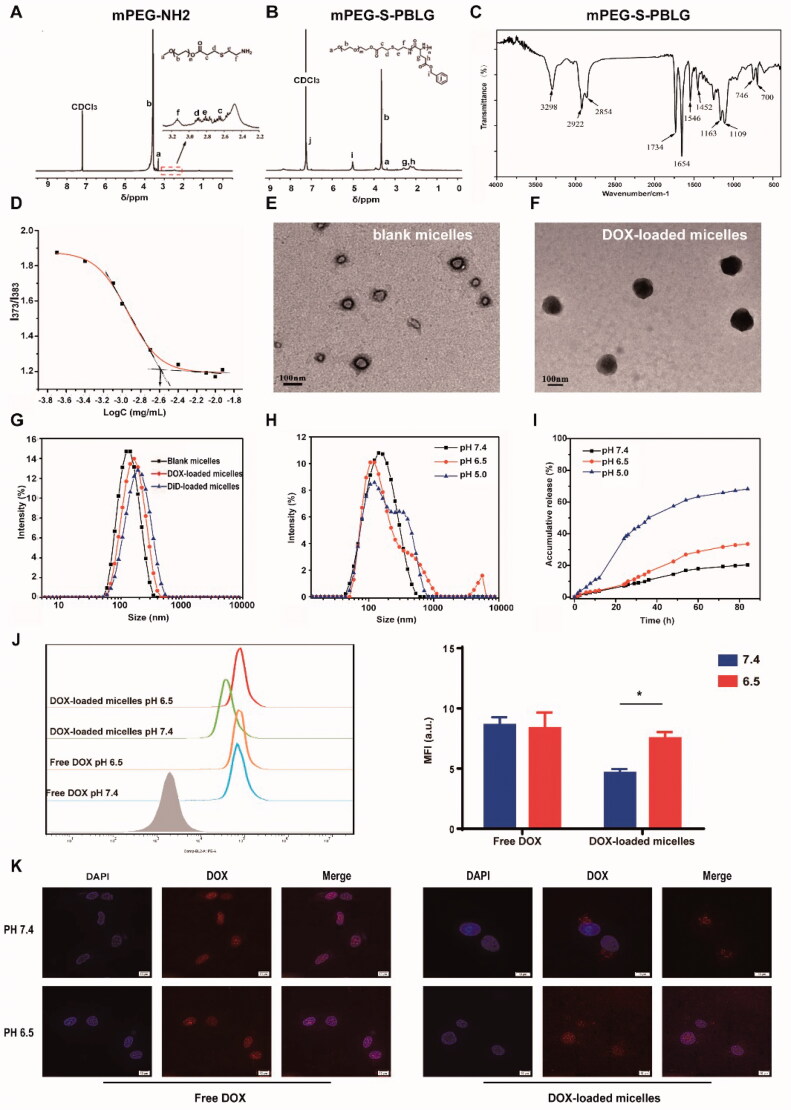
Chemical characterization, particle size, and pH-responsive properties of blank and DOX-loaded mPEG-S-PBLG micelles. ^1^H NMR spectra of mPEG-NH_2_ (A) and mPEG-S-PBLG polymer (B); (C) FT-IR spectra of mPEG-S-PBLG copolymer; (D) CMC measured by plot of the I_373_/I_383_ ratio from pyrene excitation spectra versus the concentration of polymer on a log scale. Representative TEM image of blank micelles (E) and DOX-loaded micelles (F); (G) particle size of blank and DOX/DiD-loaded micelles at pH 7.4 measured by DLS. (H) Particle size of blank micelles measured by DLS after 48-h incubation in PBS with different pH value; (I) *in vitro* drug release profile from DOX-loaded micelles at different pH value; (J) flow cytometric analysis of U-87 MG cells treated with DOX or DOX-loaded micelles at pH 7.4 and 6.5 for 6** **h. Data represent mean ± SD (*n*** **=** **3). *p** **<** **.05 analyzed by Student’s *t*-test. (K) Representative confocal image of U-87 MG cells after 6-h incubation with free DOX (5** **μM) and DOX-loaded micelles (5** **μM equivalent DOX) at pH 7.4 or 6.5 at 37** **°C. Scale bar: 10** **μm.

The primary amine could be used as the initiator to synthesize the poly(amino acid)s by the ROP of NCA monomers in a controlled way (Figure 1(A)). Cysteamine was introduced onto mPEG acrylate group by the Michael addition. In this work, the pH-sensitive copolymers (mPEG-S-PBLG) were synthesized through one-step ROP of l-BLG NCA with mPEG-S-NH_2_ as macroinitiator in DMF at 35 °C. As shown in Figure 2(A,B), the peaks of ^1^H NMR analysis proved the successful synthesis of mPEG-NH_2_ and mPEG-S-PBLG. By calculating the ratio of the integral area of peaks a–i, the polymerization degree of PBLG was 18, which was close to the theoretical value. In addition, peaks of c–f confirmed the existence of β-thiopropionate bonds. In the FT-IR spectra of mPEG-S-PBLG, there were characteristic absorption peaks of secondary amide at 1546 cm^−1^ and 1654 cm^−1^, thiopropionate at 1734 cm^−1^, 1163 cm^−1^, and 1109 cm^−1^, further confirming the successful polymerization of the copolymer (Figure 2(C)). The weight- and number-average molecular weights of mPEG-PBLG were determined to be around 17 kDa and 10.9 kDa by GPC, and the corresponding polydispersity index (PDI, Mw/Mn) was 1.55. The CMC of mPEG-S-PBLG copolymer determined using pyrene as fluorescence probe was 2.5 μg mL^−1^ (Figure 2(D)).

### Preparation of blank and DOX-loaded micelles

The amphiphilic copolymers were able to form core–shell micelles in deionized water and the DOX-loaded micelles were prepared by solvent-exchange method. The morphology of blank micelles and DOX-loaded micelles were observed under TEM (Figure 2(E,F)), and the images showed that they are spherical and uniform, with the average diameter of 60–80 nm and 100–120 nm, respectively. DLS measurement demonstrated that the average particle size of blank micelles and DOX-loaded micelles were 126 nm and 145 nm, with the PDI of 0.109 and 0.13, respectively (Figure 2(G)). Compared with the blank micelles, the particle size of DOX-loaded micelles increased slightly, indicating that DOX was effectively encapsulated. The zeta potential of PEG-S-PBLG micelles was −10.8 mV (Figure S2). The DLC and efficiency of micelles were determined by UV spectrum and the results are shown in Table 1. When the weight ratio of DOX to polymer is 2:10, the DLC was 7.8% and the DLE was 42.3%. The further increase of DOX/polymer weight ratio could not effectively improve the DLC. In addition, as shown in Figure S3, the particle size of DOX-loaded micelles kept in PBS (pH 7.4) at 4 °C for one month increased slightly and less than 30% DOX was released from DOX-loaded micelles at 4 °C for one month, which proved the colloidal stability of the micelles under neutral conditions. Overall, the mPEG-S-PBLG micelles were successfully prepared, and DOX could be effectively encapsulated into the micelles.

**Figure 3.**
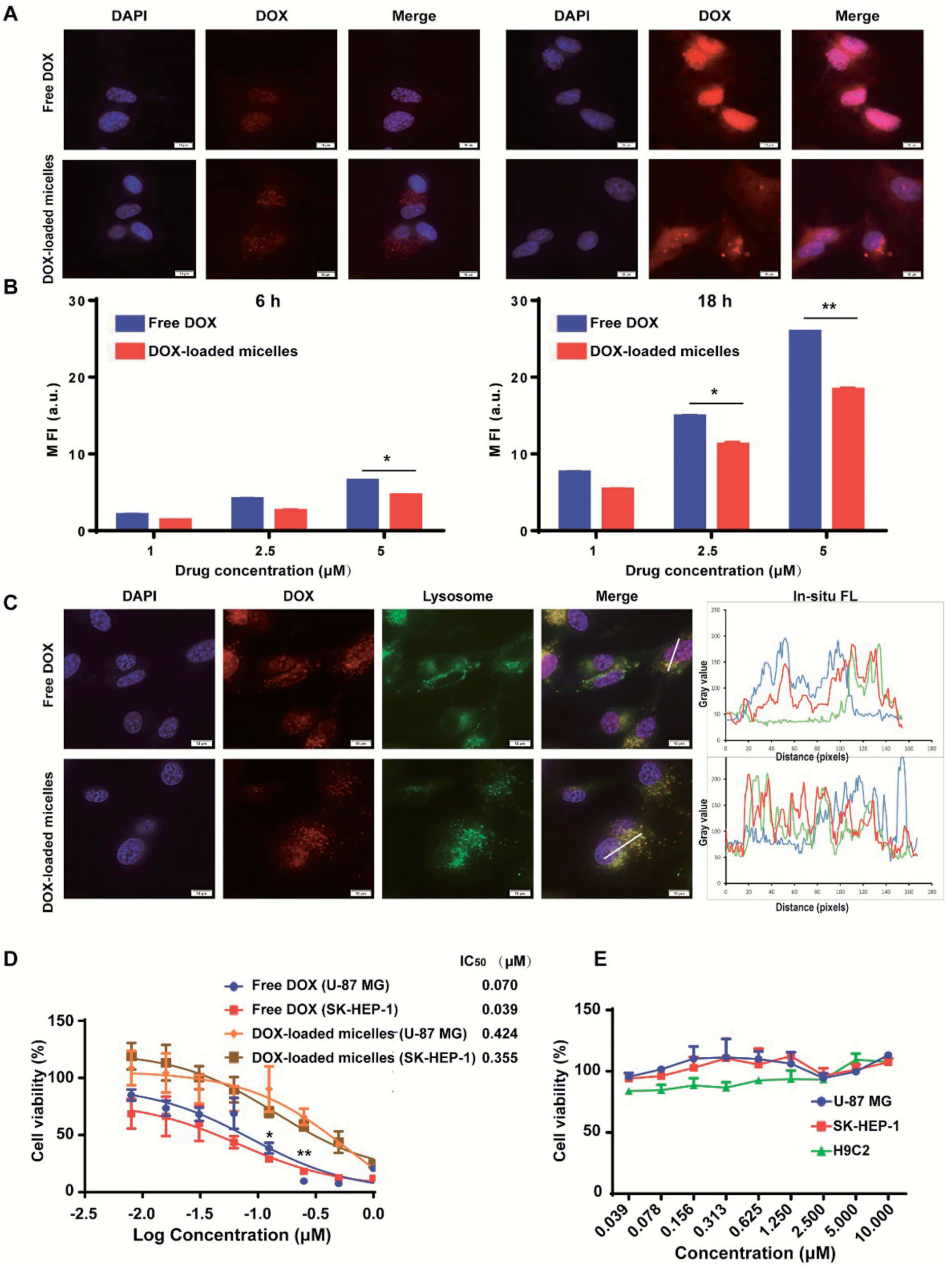
*In vitro* cellular uptake, intracellular fate, and cytotoxicity of DOX-loaded micelles in cancer cells. Confocal images (A) and flow cytometric analysis (B) of U-87 MG cells incubated with free DOX and DOX-loaded micelles (5** **μM equivalent DOX) for 6** **h and 18** **h. (C) Subcellular localization of free DOX and DOX-loaded micelles in live U-87 MG cells after 6-h incubation observed under confocal microscopy (Lyso-Tracker was used to label lysosomes) (blue line in *in situ* FL spectra represents the fluorescence of nucleus, red line represents the fluorescence of DOX and green line represents the fluorescence of lysosome). (D) Cell viability of U-87 MG human glioma cells and SK-HEP-1 human hepatocarcinoma cells after 72** **h-incubation with free DOX and DOX-loaded micelles and their IC_50_ values. (E) Cell viability of normal and cancer cells after 72-h incubation with blank micelles. Scale bar: 10** **μm. Data represent mean ± SD (*n*** **=** **3). *p** **<** **.05 **p** **<** **.01 analyzed by Student’s *t*-test.

**Table 1.** Physicochemical characterization of blank and DOX/DiD-labeled micelles.

Drug loading	Weight ratio of drug/polymer	DLC (wt%)^a^	DLE (%)^a^	Diameter (nm)^b^	Diameter (nm)^c^	PDI^c^
Blank				65	126.0	0.105
DOX	1:10	6.8	73.0			
DOX	2:10	7.8	42.3	120	144.8	0.147
DOX	3:10	7.7	27.8			
DOX	4:10	7.3	19.7			
DiD dye	2:10	7.3	39.6		172.0	0.142

^a^ Drug loading capacity was determined by ultraviolet quantitative analysis.

^b^ Average diameter of micelles was determined by TEM.

^c^ Intensity-average diameter of micelles was measured by DLS.

### pH-responsive stability and drug release

^1^H NMR data (Figure S4) of the polymer dialyzed in an acidic (pH 5.0) aqueous solution demonstrated that the peaks of PEG disappeared, indicating that the polymer chains would break under acidic conditions. The pH-responsive feature of mPEG-S-PBLG micelles was further characterized by the changes of particle size, morphology, and drug release in aqueous solution and cancer cells under different pH conditions, respectively. The tumoral acidic microenvironment and intracellular endosomes/lysosomes were simulated in PBS at pH 6.5 and pH 5.0, respectively. First, the stability of micelles in particle size at different pH value was investigated. As shown in Figure 2(H), the size of micelles remained unchanged after incubation at pH 7.4 for 48 h, while they became heterogeneous and even aggregated at pH 6.5 and pH 5.0. This indicates that the structure and integrity of the micelles could be destroyed after the β-thiopropionate linkage breaks in response to acidic environment. The morphology of micelles under different pH conditions was investigated by using TEM. As shown in Figure S5, micelles were aggregated and the morphology were seriously changed (their shape changed from round to irregular, and their edges became rough). In addition, the particle size increased after incubation at pH 6.5, which is consistent with the DLS results. Then, the drug release of DOX-loaded mPEG-S-PBLG micelles in PBS with different pH value (7.4, 6.5, and 5.0) at 37 °C was performed by the dialysis method. As shown in Figure 2(I), the release of DOX from the micelles was slow at pH 7.4, with only 20% of DOX released within 84 h; however, the release was significantly faster at pH 6.5 and pH 5.0, and the cumulative drug release rate was about 40% and 75%, respectively. In addition, the pH-responsive drug release of the micelles was further confirmed in cancer cells. The cellular uptake and distribution of DOX and DOX-loaded micelles in U-87 MG cells were observed under confocal microscopy and quantitatively analyzed by flow cytometry after 6-h incubation at different pH conditions. As shown in Figure 2(J), the cellular uptake of DOX-loaded micelles at pH 6.5 was significantly higher than that at pH 7.4. Confocal images showed that DOX-loaded micelles were more distributed in the cytoplasm and nucleus, but not in the endocytic vesicles at pH 6.5 compared with pH 7.4, indicating that DOX was widely released inside and outside cancer cells in acidic environment (Figure 2(K)). However, free DOX did not show significant changes in flow cytometry and confocal images at pH 7.4 and 6.5.

**Figure 4.**
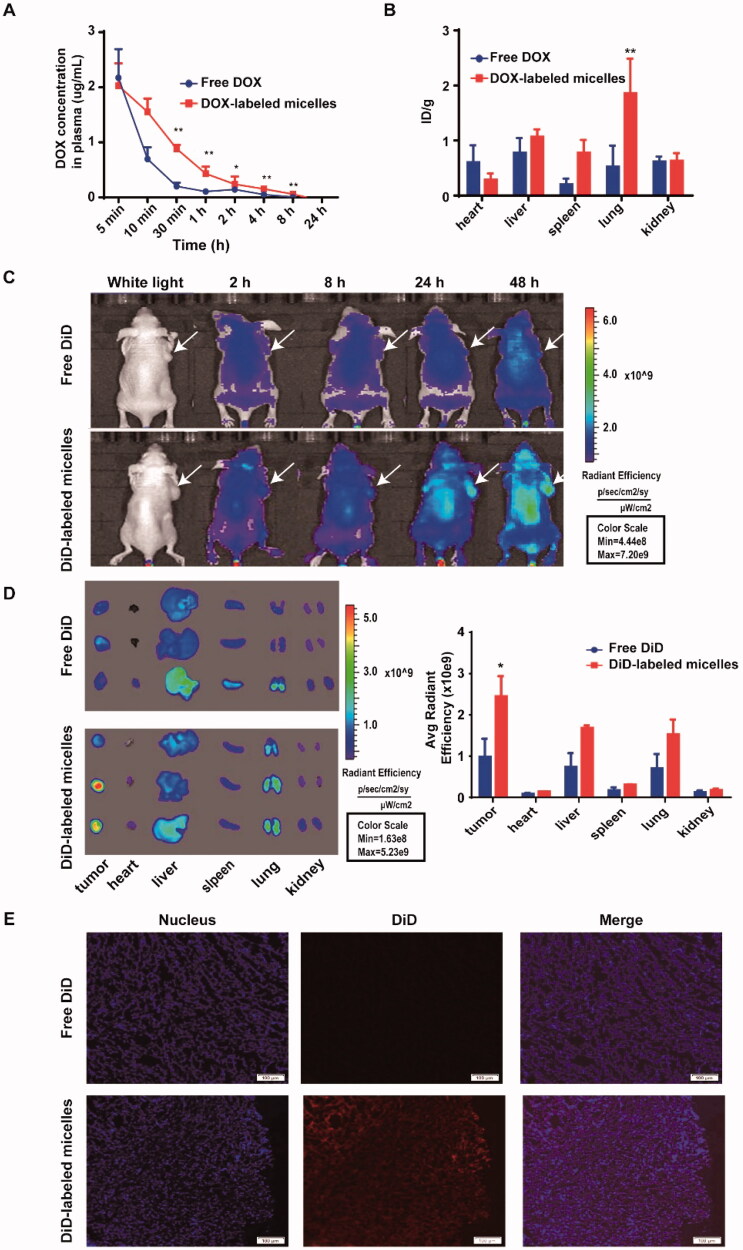
*In vivo* pharmacokinetics, biodistribution, and tumor targeting capability of DOX/DiD-labeled micelles in mice. Pharmacokinetics (A) and biodistribution data (B) of free DOX and DOX-labeled micelles given intravenously at a dose of 10** **mg kg^–1^ in BALB/c mice (*n*** **=** **5). *In vivo* (C) and *ex vivo* (D) NIRF optical images of U-87 MG tumor-bearing mice injected intravenously with free DiD dye and DiD-labeled micelles. At 48** **h post-injection, tumors and major organs were harvested for *ex vivo* imaging, and the corresponding fluorescence intensities were quantified (*n*** **=** **3). (E) The microscopic distribution of free DiD and DiD-labeled micelles in tumor sections. The nuclei were stained with DAPI (blue). Scale bar: 100** **μm. Data represent mean ± SD. *p** **<** **.05 **p** **<** **.01 analyzed by Student’s *t*-test or two-way ANOVA.

**Figure 5.**
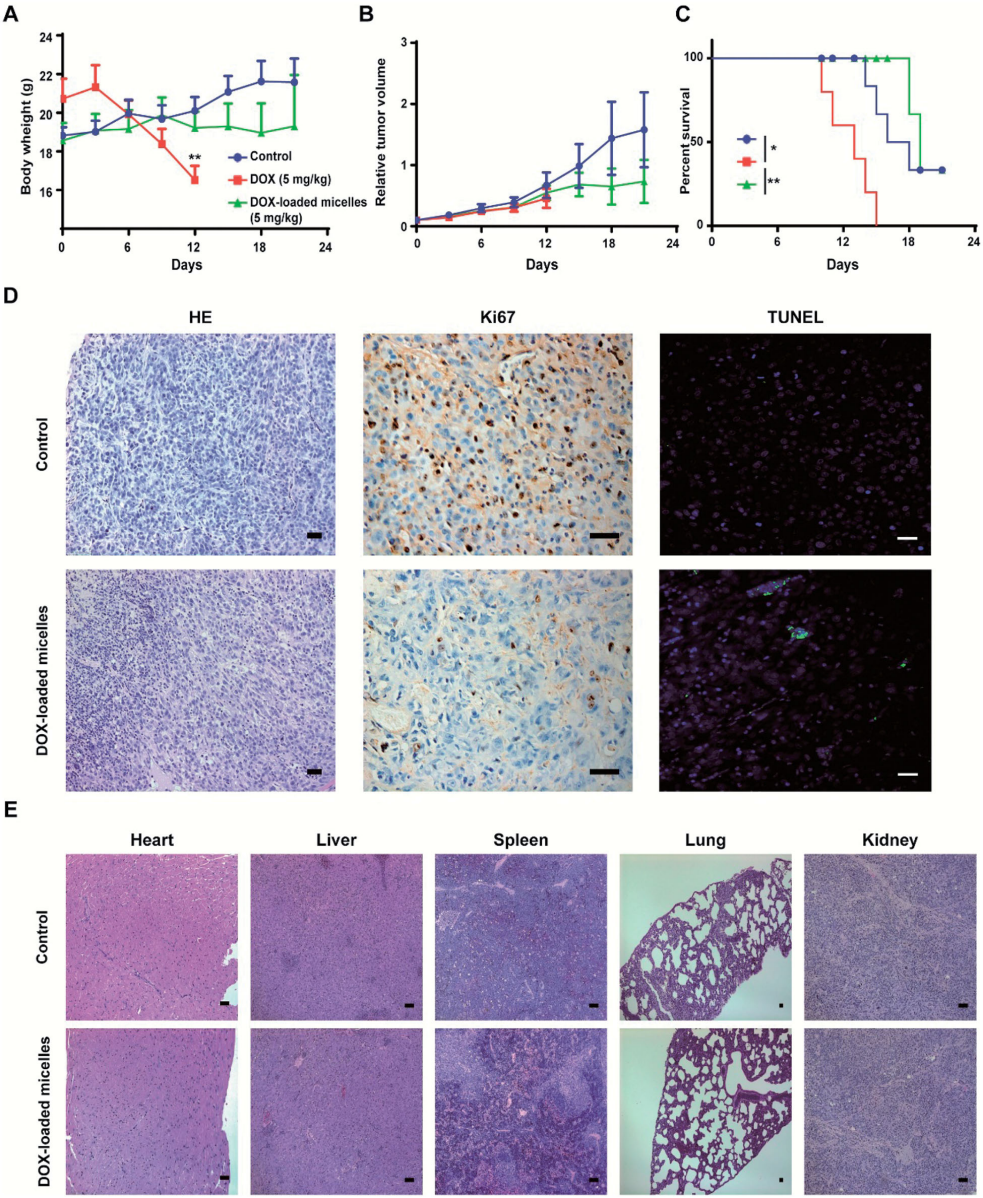
Therapeutic efficacy and toxicity profiles of DOX-loaded micelles in U-87 MG tumor bearing mice. Body weight changes (A), Kaplan–Meier’s survival curve (B), *in vivo* tumor growth inhibition (C) of U-87 MG tumor bearing mice after intravenous treatment of different DOX formulations (5** **mg kg^–1^ each dose for a total of seven doses) (*n*** **=** **5). Data are expressed as means ± SD. *p** **<** **.05, **p** **<** **.01 analyzed by two-way ANOVA or log rank test. (D) Histology (HE) and immunohistochemical staining (Ki67 and TUNEL) of tumor tissues. Scale bar: 100** **μm. (E) Histological analysis (HE staining) of other major organs excised from treated mice. Scale bar: 200** **μm.

### Cellular uptake and intracellular distribution

The uptake and intracellular fate of DOX-loaded micelles in U-87 MG cells were observed by confocal microscopy and flow cytometry. After 6-h incubation, both free DOX and DOX-loaded micelles were able to be internalized into the cells. However, free DOX was mainly distributed in the nuclear region, while DOX-loaded micelles were mainly found in red fluorescent spots in the perinuclear area of cytoplasm (Figure 3(A)). In addition, the cellular uptake of DOX-loaded micelles in U-87 MG cells was further quantitatively analyzed by flow cytometry. In accordance with the results observed by CLSM, the uptake of DOX-loaded micelles was concentration- and time-dependent, and their uptake rate was slightly lower than that of free DOX (Figure 3(B)). Then, the subcellular distribution of DOX-loaded micelles upon internalization was further confirmed by lysosome co-localization experiment. Confocal microscopy (Figure 3(C)) demonstrated that after 6-h incubation with U-87 MG cells, most of DOX-loaded micelles (red) were co-localized with lysosomal compartment (green), as shown by yellow fluorescence in the merged images, while most of free DOX was mainly distributed in the nucleus. After 18-h incubation, most of the fluorescence from DOX-loaded micelles was redistributed in the cytoplasm and nucleus, suggesting that DOX can escape from the endosomes/lysosomes through pH-responsive release process (Figure 3(A)).

### *In vitro* cytotoxicity

The *in vitro* cytotoxicity of DOX-loaded micelles against cancer cells was evaluated by CCK-8 assay. As shown in Figure 3(D), DOX-loaded micelles exhibited concentration-dependent cytotoxicity against both U-87-MG and SK-HEP-1 cancer cells after 72-h incubation, but their IC_50_ values were about 6–9 times higher than those of free DOX, respectively. In addition, no obvious cytotoxicity was observed in these cancer cells as well as H9C2 normal cells after 72-h incubation with blank micelles at the tested concentrations (Figure 3(E)), indicating their good biocompatibility.

### Pharmacokinetics and biodistribution

The pharmacokinetics and biodistribution profiles of DOX-loaded micelles were determined in tumor-free female BALB/c nude mice. According to the standard curve of DOX in blood (Figure S6), Figure 4(A) illustrates the time-dependent curve of DOX concentration in the blood of mice receiving a single dose of free DOX or DOX-loaded micelles (10 mg kg^−1^). The corresponding pharmacokinetic parameters are summarized in Table S1. The results showed that DOX-loaded micelles had longer half-life (*t*_1/2_), higher area under curve (AUC), greater mean residence time (MRT), and lower clearance rate (CL) than free DOX. This suggests that the micellar formulation can prolong the blood circulation and reduce the elimination rate of delivered drugs, which may promote their deposition in tumor tissues. The biodistribution of DOX-loaded micelles in major organs at 24 h post-injection was also measured. As shown in Figure 4(B), compared with free DOX, the micellar formulation significantly reduced the distribution of DOX in the heart, which means that the micelles may reduce DOX-induced cardiotoxicity. In addition, the distribution of DOX-loaded micelles in lung, liver, and spleen was relatively higher than free DOX, which may be due to the nonspecific clearance of reticuloendothelial system (RES).

**Figure 6.**
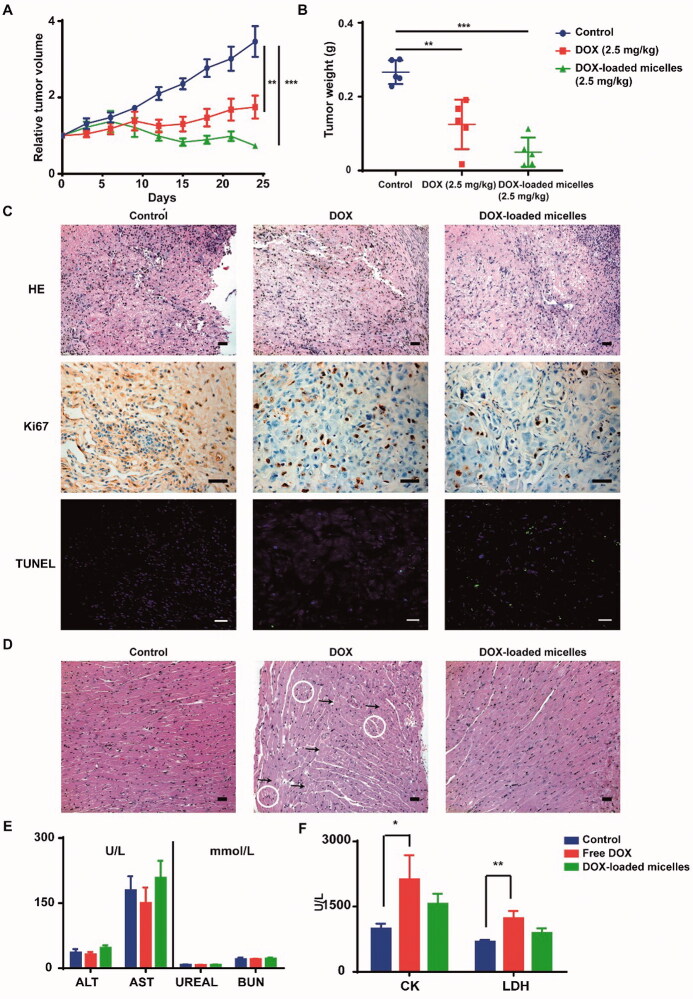
Therapeutic efficacy and toxicity profiles of DOX-loaded micelles in SK-HEP-1 tumor bearing mice. *In vivo* tumor growth inhibition (A) and tumor weight (B) of SK-HEP-1 tumor bearing mice after intravenous administration of different DOX formulations (2.5** **mg kg^–1^ each dose for a total of seven doses) (*n*** **=** **5).(C) Histology (HE) and immunohistochemical staining (Ki67 and TUNEL) of tumor tissues. Scale bar: 100** **μm. (D) Histological images (HE staining) of hearts excised from treated mice. The intracytoplasmic vacuolation and the degradation of muscle fibers were marked by white circle and black arrows, respectively. Scale bar: 100** **μm. (E) Serum chemistry analysis of mice treated with different DOX formulations, including hepatic function (ALT and AST), renal function (UREAL and BUN), and cardiac toxicity (F, CK and LDH) (*n*** **=** **5). Data are expressed as means ± SD. *p** **<** **.05, **p** ***<*** **.01, ***p** ***<*** **.001 analyzed by one-way ANOVA or two-way ANOVA.

In another set of experiment, the real-time biodistribution and tumor targeting capability of fluorescent dye DiD-labeled micelles were visualized by NIRF optical imaging in U-87 MG tumor bearing mice. As shown in Figure 4(C), DiD-labeled micelles were able to gradually accumulate to the tumor site, whereas no obvious uptake of free DiD was observed in the tumor. *Ex vivo* images at 48 h post-injection further demonstrated that DiD-labeled micelles could preferentially accumulate into the tumor, with stronger fluorescence intensity than other organs (Figure 4(D)). Quantitative analysis showed that the mean fluorescence intensity of DiD-labeled micelles in tumor tissues was about 2.5-folds higher than that of free DiD. Furthermore, microscopic images demonstrated that DiD-labeled micelles distributed throughout the tumor tissue and their fluorescence signals in cancer cells were significantly higher than that of free DiD (Figure 4(E)).

### *In vivo* therapeutic study

The anti-tumor efficacy and toxicity of DOX-loaded micelles were first evaluated in subcutaneous xenograft model of U-87 MG glioblastoma. DOX-loaded micelles and free DOX were intravenously administered every other day at the dose of 5 mg kg^−1^ for seven times (cumulative dose of 35 mg kg^−1^), respectively. Unexpectedly, the body weight of mice treated with free DOX continued to drop rapidly, and all mice died on the 15th day. However, the mice treated with DOX-loaded micelles were well tolerated with only slight weight loss (Figure 5(A)). Compared with the control group, DOX-loaded micelles effectively inhibited the tumor growth (Figure 5(B)) and prolonged the survival time (Figure 5(C)). Hematoxylin and eosin (HE) staining of tumor tissue showed widespread nuclear pyknosis and chromatin condensation in the DOX-loaded micelles group, but not in the control group (Figure 5(D)). In addition, the immunohistochemical staining of tumor tissue in the DOX-loaded micelles group showed a significant decrease in Ki-67 positive cells and a significant increase in TUNEL positive cells (Figure 5(D)) compared to that in the control group. The results from HE staining of major organs demonstrated that there was no obvious change in the histology of heart, liver, spleen, lung, and kidney from mice treated with DOX-loaded micelles (Figure 5(E)).

In addition, the therapeutic efficacy and safety profiles of DOX-loaded micelles were also evaluated in another subcutaneous xenograft model of SK-HEP-1 liver cancer. In order to avoid the lethal toxicity caused by DOX, each dose was adjusted to 2.5 mg kg^−1^ in this experiment. Compared to the saline control, both free DOX and DOX-loaded micelles were able to significantly inhibit the tumor growth (*p*< .05, Figure 6(A)), with lower tumor weight at the end of experiment and higher survival rate (Figure 6(B) and Figure S7). However, the micellar formulation exhibited better tumor growth inhibition than free DOX. Histological and immunohistochemical staining of tumor tissues demonstrated that more necrotic cells, less Ki-67 positive cells, and more TUNEL positive cells were observed in the DOX-loaded micelles group compared to the saline control and free DOX group (Figure 6(C)). The results from HE staining of heart tissues showed that the intracytoplasmic vacuolation and the degradation of muscle fibers (represented by the wavy shape of muscle fibers) were obviously observed in mice treated with free DOX (Figure 6(D)). On the contrary, there was no significant histological change in the heart of mice treated with DOX-loaded micelles, suggesting that the micellar formulation could attenuate DOX-induced cardiomyopathy. At the end of the experiment, the blood was collected for serum chemistry analysis. The results showed that the hepatic and renal function panels including ALT, AST, UREAL, and BUN were all within the normal range in all the treatment groups (Figure 6(E)). However, the representative indexes of myocardial function such as serum CK and LDH levels in the free DOX group were significantly higher than those in the control group, respectively (*p*< .05, *p*< .01), while the micellar formulation greatly inhibited the elevation of CK and LDH induced by DOX (Figure 6(F)).

## Discussion

DOX is an effective chemotherapeutic drug commonly used in a variety of cancers. However, patients treated with DOX are often unable to tolerate severe side effects, resulting in a decline in long-term clinical efficacy (Alyane et al., 2016). Cardiotoxicity is one of the most common side effects of DOX, which may lead to irreversible cardiomyopathy and consecutive congestive heart failure (Fang et al., 2018). Polymeric micelles have been reported as promising carriers of many anticancer drugs due to their function as sustained, controlled, and targeted drug delivery systems (Li et al., 2010). The micelles, commonly composed of amphiphilic copolymer, were able to self-assemble with core/shell structure in aqueous solution and DOX were efficiently encapsulated in the core of the micelles to achieve better solubility and bioavailability. However, there are still challenges in terms of early drug release in blood circulation and sustained drug release in tumor sites (Zhou et al., 2020).

Since the pH value of tumor sites (6.5–7.0) is lower than that of normal tissues (∼7.4), pH-responsive polymeric micelles are expected to increase drug accumulation in tumor sites and reduce drug leakage to normal organs and tissues (Gannimani et al., 2020). In this study, the pH-sensitive copolymers (mPEG-S-PBLG) were synthesized through one-step ROP of l-BLG NCA with mPEG-S-NH_2_ as macroinitiator. The acid-labile β-thiopropionate linkage was introduced in the backbone of mPEG-PBLG copolymer through thiol-ene click reaction, acting as a pH-responsive ‘ON/OFF’ switch for triggering micelle destruction and DOX release. It is well known that the physicochemical characteristics of micelles, such as CMC, shape, and size, are the critical factors affecting their circulation, biodistribution, cellular internalization, and tissue retention *in vivo* (Finbloom et al., 2020; Hwang et al., 2020). The CMC of mPEG-S-PBLG copolymer was 2.5 μg mL^−1^, indicating its excellent micelle-forming property. TEM images and DLS results showed that the blank micelles and DOX-loaded micelles were spherical and uniform. It was not surprising that the size of micelles measured by DLS was slightly larger than that measured by TEM, which is probably because TEM samples are usually prepared in a dry state while the copolymer may swell in aqueous solutions measured by DLS (Wei et al., 2016). The antifouling properties of PEG shell, optimal shape and size of mPEG-S-PBLG micelles may reduce the protein adsorption, prolong the blood circulation, and efficiently deliver anti-cancer drugs into the tumor site through the EPR effect (Zhou et al., 2020). Furthermore, to ensure a sufficient dose of drugs to be delivered to tumor sites, an ideal drug delivery system should achieve high DLC as well as DLE. DOX and polymer were mixed at different ratio and better DLC and DLE were achieved when the weight ratio of DOX to polymer was 2:10. The further increase of DOX/polymer weight ratio could not improve the DLC, which is probably because that the loading capacity reached the plateau at the weight ratio of 2:10, and the addition of more DOX may induce aggregation and precipitation, resulting in the decrease in DLC and DLE. The cellular uptake was qualitatively and quantitatively studied by using CLSM and flow cytometry. Interestingly, some dot-shape fluorescent foci were observed at the perinuclear region of the cytoplasm in the cells treated with DOX-loaded micelles, but not in the cells treated with free DOX. Similar phenomenon was also observed by Xiao et al. in the Raji cells incubated with DOX-loaded PEG-CA (cholic acid) micelles (Xiao et al., 2011). This is probably because the entry of DOX is mainly through the diffusion of small molecules while the internalization of DOX-loaded micelles is mainly mediated by endocytosis (Finbloom et al., 2020). The lysosome staining experiment demonstrated that the DOX-loaded micelles were initially co-localized with the lysosome at 6** **h, subsequently released DOX from the lysosome, and finally reached the nuclear region at 18** **h, indicating their pH-responsive drug release characteristics. Flow cytometry analysis demonstrated that the cellular uptake of DOX-loaded micelles seemed to be slightly slower than that of free DOX. That is probably because DOX is an amphiphilic small molecule which can enter tumor cells via free diffusion and phagocytosis, but the micellar formulation needs to go through the endocytosis process before entering a lysosome in which the loaded DOX is released (Duan et al., 2018).

Compared to other pH responsive linkages, β-thiopropionate linkage can undergo hydrolysis in mildly acidic environment at a relatively slow rate, so controlled drug release in a sustained manner is expected (Schoenmakers et al., 2004; Pramanik et al., 2016; Qiu et al., 2016). Additionally, this linkage can be synthesized by a simple, facile thiol methacrylate Michael addition reaction (Schoenmakers et al., 2004; Pramanik et al., 2016; Qiu et al., 2016). We have fabricated the micelles by incorporating the β-thiopropionate moiety into the polymer that can be cleaved under acidic TME, thus achieving controlled drug release from the micelles. As β-thiopropionate linkage is cleavable at pH 5.5–6.5, the DOX-loaded micelles were supposed to be stable during the blood circulation (pH 7.4) and disrupted to release drug subsequently in tumor sites (pH 6.5–7.0) (Pramanik et al., 2016). For this reason, the size and morphology of micelles remained stable at pH 7.4, but changed significantly at pH 6.5 and pH 5.0, which indicates that the structure and integrity of the micelles could be destroyed after the β-thiopropionate linkage breaks in response to acidic environment. Furthermore, the drug release rate of micelles was relatively slow under neutral conditions, but can be significantly accelerated under acidic conditions, showing good pH-responsiveness. It is reported that some pH-responsive linkages, such as acetal linkages, are highly sensitive to acid condition, resulting in rapid breakdown of linkage and burst release of drugs from NPs (more than 50%) at lower pH in the first 24** **h (Kim et al., 2009; Shim & Kwon, 2012; Tu et al., 2013). It should be noted that compared with other pH responsive NPs, the initial drug release from mPEG-S-PBLG micelles in response to pH stimulus was relatively slow (less than 20% and 40% drugs were released at pH 6.5 and 5.0 during 24** **h) due to the lower degradation rate of β-thiopropionate linkage under acidic conditions than other acid-labile functional groups (Chen et al., 2016; Pramanik et al., 2016). The micellar formulation also increased the cellular uptake of DOX at pH 6.5 due to the cleavage of β-thiopropionate linkage, allowing the extensive DOX release. Based on this pH-responsive and sustained drug release property, the drug-loaded micelles might present desirable inhibition of tumor growth while reducing side effects (Pramanik et al., 2016; Jia et al., 2020). The *in vitro* cytotoxicity data showed that blank micelles had no obvious toxicity in both cancer cells and normal cells, indicating each unit of the micelles-forming polymers (including PEG and PBLG) are nontoxic and biocompatible. On the contrary, DOX-loaded micelles exhibited concentration-dependent cytotoxicity against both U-87 MG cells and SK-HEP-1 cells, suggesting that DOX could be released from the micelles and effectively inhibited tumor proliferation. Notably, the IC_50_ value of DOX-loaded micelles was slightly higher than that of free DOX. This is probably because the small molecule drug DOX can spread rapidly into the nucleus to damage DNA, while the micelles need to enter the lysosome by endocytosis and then continuously release DOX in a pH-responsive manner (Duan et al., 2018).

It is well known that longer blood circulation time, better tumor targeting, and more drug accumulation in the tumor site can improve the therapeutic effect and reduce the toxicity (Lu et al., 2018; Zhou et al., 2018). As expected, the micellar formulation did increase the residence time of DOX in the blood compared with free DOX. This may be due to the fact that PEG on hydrophilic surface can counter hydrophobic and electrostatic interactions between micelles and plasma proteins or macrophages (Kooijmans et al., 2016), thus prolonging the blood circulation time of micelles. Interestingly, biodistribution studies showed that the micelles could reduce the distribution of DOX in the heart to a certain extent, showing the potential to reduce DOX related cardiotoxicity. In addition, the micelles could enhance the accumulation of DOX into the lung, suggesting the micellar formulation may have therapeutic effects on lung metastasis. Furthermore, the *in vivo* and *ex vivo* NIRF optical images and immunofluorescence images of tumor slices demonstrated that the micelles could preferentially accumulate and penetrate into the tumor tissue due to the EPR effect, and release drugs in response to acidic TME.

The anti-tumor efficacy and toxicity profiles of DOX-loaded micelles were evaluated in two different subcutaneous xenograft models (U-87 MG glioblastoma and SK-HEP-1 hepatoma). As expected, DOX-loaded micelles exhibited better tumor inhibition and survival advantages compared with the equivalent dose of free DOX, accompanied by decreased proliferation (Ki67 positive) and increased apoptosis (TUNEL positive) of cancer cells. The improved efficacy could be explained by the enhanced tumor targeting capability of the micellar formulation, which facilitated more distribution and pH-responsive release of DOX in the tumor site. There is evidence that the severe cardiotoxicity of DOX limits its clinical application to a great extent (Fang et al., 2018). As demonstrated in our study, free DOX exhibited significant systemic toxicity (body weight loss and even animal death) and cardiotoxicity (cardiac histopathological abnormalities, elevated serum LDH, and CK levels). Very importantly, the micellar formulation significantly reduced systemic and cardiac toxicity caused by DOX, which may be due to the prolonged blood circulation, enhanced accumulation and pH-responsive release in the tumor. Moreover, the cleavage of β-thiopropionate at acidic TME could trigger size increase and aggregation of micelles by increasing the hydrophobicity of DOX, which is conducive to enhance the retention of micelles in tumor and reduce their distribution in heart (Zhang et al., 2020). In addition, no obvious pathological alterations of major organs such as liver, spleen, lung, and kidney were observed in mice treated with DOX-loaded micelles, and their serum chemistry indexes including hepatic and renal function panels (ALT, AST, UREAL, and BUN) were within the normal range, indicating the micellar formulation had good biocompatibility and safety. Taken together, our data have demonstrated that the newly developed pH-responsive mPEG-S-PBLG micelles are able to selectively deliver and release anti-cancer drugs such as DOX to tumor sites, thus improving therapeutic efficacy and reducing toxicity.

## Conclusions

In summary, we have successfully developed a novel class of tumor pH-responsive mPEG-S-PBLG micelle platform for the targeted delivery of chemotherapeutics. The micelles were able to efficiently encapsulate DOX, prolong the blood circulation time, facilitate the tumor accumulation via the EPR effect, and programmatically release the drug in response to acidic TME, resulting in improved anti-tumor efficacy and lower systemic toxicity. It was also noted that the pH-responsive micellar formulation significantly reduced the distribution of DOX in the heart tissue, and attenuated DOX-associated cardiomyopathy.

## Supplementary Material

Supplemental Material
