# Supplementary material for: Targeted delivery by pH-responsive mPEG-S-PBLG micelles significantly enhances the anti-tumor efficacy of doxorubicin with reduced cardiotoxicity
Source: Drug Deliv. 2021 Nov 29;28(1):2495–509. doi: 10.1080/10717544.2021.2008052 (PMC8635546; doi:10.1080/10717544.2021.2008052)
Supplement: Supplemental Material [file IDRD_A_2008052_SM2437.docx]

**Supporting information**

**Targeted Delivery by pH-Responsive mPEG-S-PBLG Micelles Significantly Enhances the Anti-tumor Efficacy of Doxorubicin with Reduced Cardiotoxicity**

Qiyi Feng ^a,c,1^, Junhuai Xu ^b,1^, Xinyi Liu ^a,c^, Haibo Wang ^b^ , Junjie Xiong ^d,*^, and Kai Xiao ^a,c*^

^a^ Precision Medicine Research Center and National Clinical Research Center for Geriatrics, West China Hospital, Sichuan University, Chengdu, 610041, China

^b^ College of Biomass Science and Engineering, Sichuan University, Chengdu, 610041, China

^c^ National Chengdu Center for Safety Evaluation of Drugs, West China Hospital, Sichuan University, Chengdu, 610041, China

^d^ Department of Pancreatic Surgery, West China Hospital, Sichuan University, Chengdu, 610041, China

^1^ The authors contributed equally in the work.

*****Correspondence:**

Kai Xiao, E-mail: [xiaokaikaixiao@scu.edu.cn](mailto:xiaokaikaixiao@scu.edu.cn); Junjie Xiong, E-mail: junjiex2011@126.com.


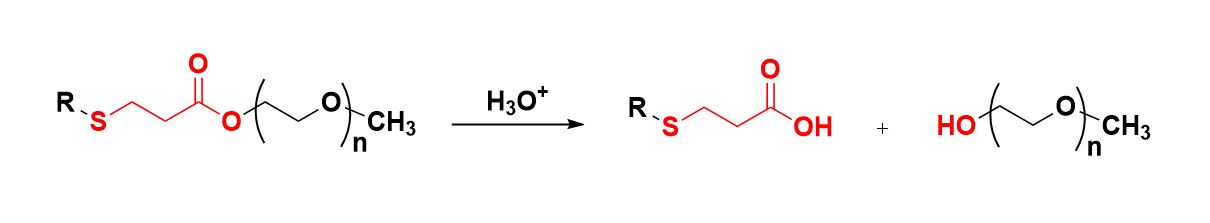
**Figure S1.** The pH responsive mechanism of β-thiopropionate linkage in mPEG-S-PBLG copolymer.

**
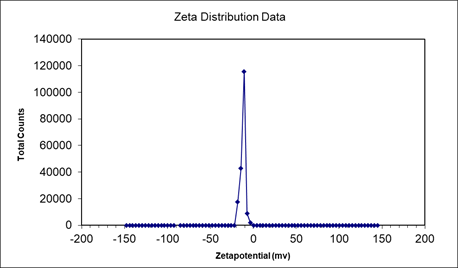
**

**Figure S2.** The zeta potential of mPEG-S-PBLG micelles.


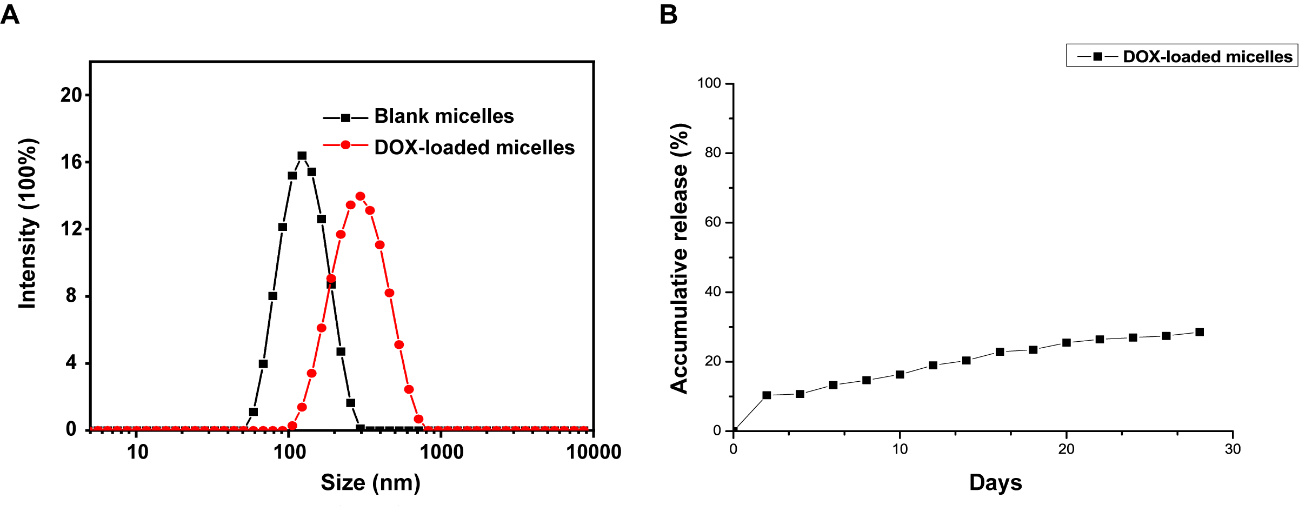
**Figure S3**. (A) Particle size of blank and DOX-loaded micelles kept in PBS (pH 7.4) at 4 ℃ for 30 days. (B) *In vitro* drug release profile from DOX-loaded micelles at 4℃ for 28 days.

**

**

**Figure S4**. ^1^H NMR spectra of mPEG-S-PBLG after dialysis against deionized water at pH 5.0 for 2 days.

**
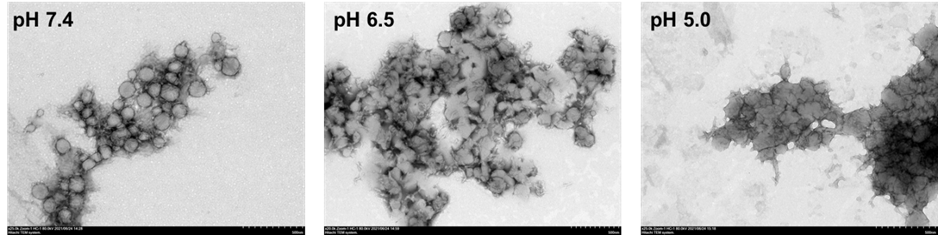
**

**Figure S5**. Representative TEM images after exposure of blank micelles on pH 7.4 (left), pH 6.5 (middle) or pH 5.0 (right) for 6 hours.


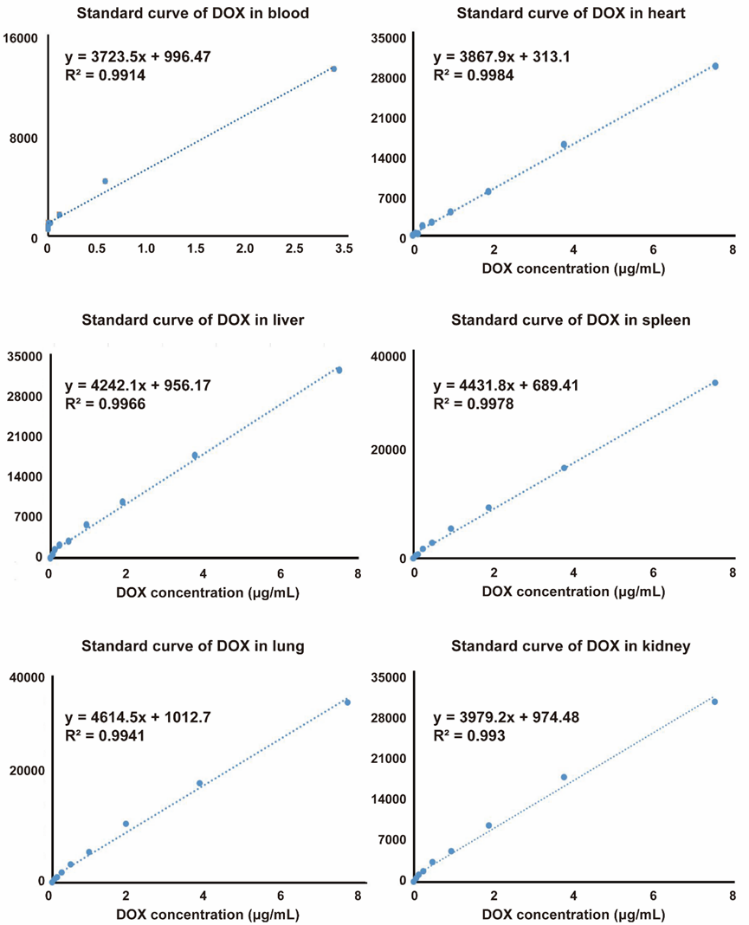


**Figure S6**. The standard curves of DOX in the pharmacokinetic study.


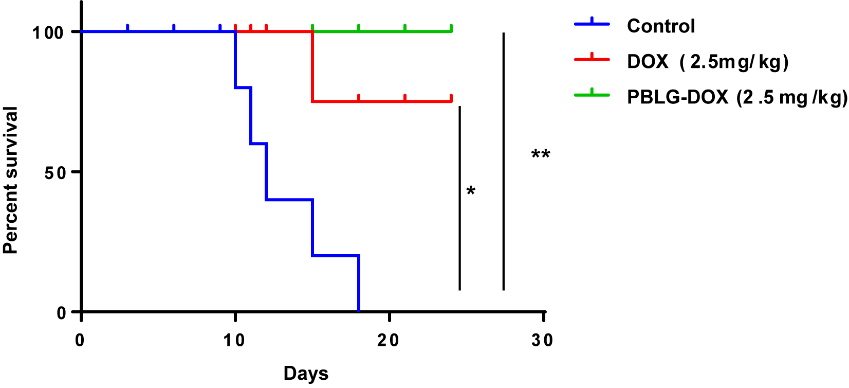
**Figure S7**. Kaplan-Meier survival curve of SK-HEP-1 tumor bearing mice after intravenous treatment of different DOX formulations. *P < 0.05 **P < 0.01 analyzed by log rank test.

**Table S1**. Pharmacokinetic parameters of BALB/c mice after intravenous administration of DOX and DOX-loaded micelles at the dose of 10 mg DOX/kg (n = 5)

| Parameters | Free DOX | DOX-loaded micelles |
| --- | --- | --- |
| t_1/2β_(h) | 1.240 ± 0.32 | 2.025 ± 0.17 |
| CL (L h^-1^ kg^-1^) | 6.118 ± 1.82 | 3.704 ± 0.63^*^ |
| AUC_0-∞_ (h ug mL^-1^) | 1.333 ±0 .33 | 2.463 ± 0.50 |
| MRT_0-∞_ (h) | 1.596 ± 0.57 | 2.772 ± 0.55^*^ |

**Notes:** * *P*＜0.05

**Abbreviations:** t_1/2β_, Elimination Half-life; CL, clearance rate; AUC, area under the curve; MRT, Mean Retention Time
